# Glycoprotein G-protein Coupled Receptors in Disease: Luteinizing Hormone Receptors and Follicle Stimulating Hormone Receptors

**DOI:** 10.3390/diseases8030035

**Published:** 2020-09-15

**Authors:** Duaa Althumairy, Xiaoping Zhang, Nicholas Baez, George Barisas, Deborah A. Roess, George R. Bousfield, Debbie C. Crans

**Affiliations:** 1 Cell and Molecular Biology Program, Colorado State University, Fort Collins, CO 80523, USA; dalthumairy@kfu.edu.sa (D.A.); George.Barisas@colostate.edu (G.B.); 2 Department of Biological Sciences, King Faisal University, Al-Ahsa 31982, Saudi Arabia; 3 Department of Chemistry, Colorado State University, Fort Collins, CO 80523, USA; Sam.Zhang2@colostate.edu (X.Z.); nickbaez@rams.colostate.edu (N.B.); 4 Department of Biomedical Sciences, Colorado State University, Fort Collins, CO 80523, USA; deborah.roess@colostate.edu; 5 Department of Biological Sciences, Wichita State University, Wichita, KS 67260, USA; george.bousfield@wichita.edu

**Keywords:** luteinizing hormone receptor, follicle-stimulating hormone receptor, hormones

## Abstract

Signal transduction by luteinizing hormone receptors (LHRs) and follicle-stimulating hormone receptors (FSHRs) is essential for the successful reproduction of human beings. Both receptors and the thyroid-stimulating hormone receptor are members of a subset of G-protein coupled receptors (GPCRs) described as the glycoprotein hormone receptors. Their ligands, follicle-stimulating hormone (FSH) and luteinizing hormone (LH) and a structurally related hormone produced in pregnancy, human chorionic gonadotropin (hCG), are large protein hormones that are extensively glycosylated. Although the primary physiologic functions of these receptors are in ovarian function and maintenance of pregnancy in human females and spermatogenesis in males, there are reports of LHRs or FSHRs involvement in disease processes both in the reproductive system and elsewhere. In this review, we evaluate the aggregation state of the structure of actively signaling LHRs or FSHRs, their functions in reproduction as well as summarizing disease processes related to receptor mutations affecting receptor function or expression in reproductive and non-reproductive tissues. We will also present novel strategies for either increasing or reducing the activity of LHRs signaling. Such approaches to modify signaling by glycoprotein receptors may prove advantageous in treating diseases relating to LHRs or FSHRs function in addition to furthering the identification of new strategies for modulating GPCR signaling.

## 1. Introduction

In both men and women, signal transduction by luteinizing hormone receptors (LHRs or LHRCGs; LHRs will be used in this review) and follicle-stimulating hormone receptors (FSHRs) is essential for successful reproduction. Both receptors are members, together with the thyroid-stimulating hormone receptor, of a subset of G-protein coupled receptors (GPCRs) described as the glycoprotein hormone receptors. In addition, their ligands, follicle-stimulating hormone (FSH), luteinizing hormone (LH), and a structurally related hormone produced in pregnancy, human chorionic gonadotropin (hCG), are large protein hormones that are extensively glycosylated. Although the primary physiologic functions of these receptors are in ovarian function and maintenance of pregnancy in human females and spermatogenesis in males, there are reports of LHR or FSHR involvement in disease processes both in the reproductive system and elsewhere. In this review, we will consider the structure of actively signaling LHRs or FSHRs as dimers or larger oligomers and their functions in reproduction, as well as summarizing disease processes related to receptor mutations affecting receptor function or expression in reproductive and non-reproductive tissues. We will also discuss novel strategies for either increasing or reducing the activity of LHRs, which may prove advantageous in treating diseases relating to LHRs or FSHRs function.

## 2. LHR and FSHR Structure and Function

### 2.1. Luteinizing Hormone Receptors (LHRs)

LHRs are critical to reproduction in both mammalian sexes [1]. The receptors are mainly present in gonadal cells, including testicular Leydig cells in men [2] and target cells in the follicle and corpus luteum of the ovary in women. In human follicles, LHRs are found in the theca, interstitial, and granulosa cells of the follicle [3]. LHRs have also been identified in a number of non-gonadal cells, including the human uterus and human skin [1]. In most, if not all cases, the function of LHRs in these tissues is not known.

In males, LHRs are essential for the development of the external male genitalia; LHRs expressed during fetal life are activated by maternal human chorionic gonadotropin (hCG). During puberty, LHRs, responding to pituitary LH, is involved in the production and secretion of androgens, which induce the development of the secondary sex characteristics. In the human testis, where LHRs in Leydig cells and FSHRs in Sertoli cells have been identified using immunohistochemistry [4], LHRs, stimulated by pituitary LH [2,5,6], are necessary for sperm maturation. In females, the LHRs are found in theca cells and differentiated granulosa cells in the ovarian follicle and on luteal cells of the corpus luteum [1]. LHRs expressed in follicular granulosa and theca cells are necessary for the development of the follicle, enhanced steroidogenesis, and ovulation [2,7]. Active LHRs on luteal cells in the corpus luteum, a hormone-producing gland formed from the post-ovulatory follicle, bind LH to maintain synthesis and secretion of estrogen and progesterone by steroidogenic cells. During female fetal development, LHRs are not detectable; receptor expression begins, and only at very low levels, in neonates [5]. In puberty, a pituitary LH surge causes the initiation of the first ovarian cycle, which, in this and subsequent cycles, ends with menstruation in the absence of pregnancy [8].

**Figure 1:**
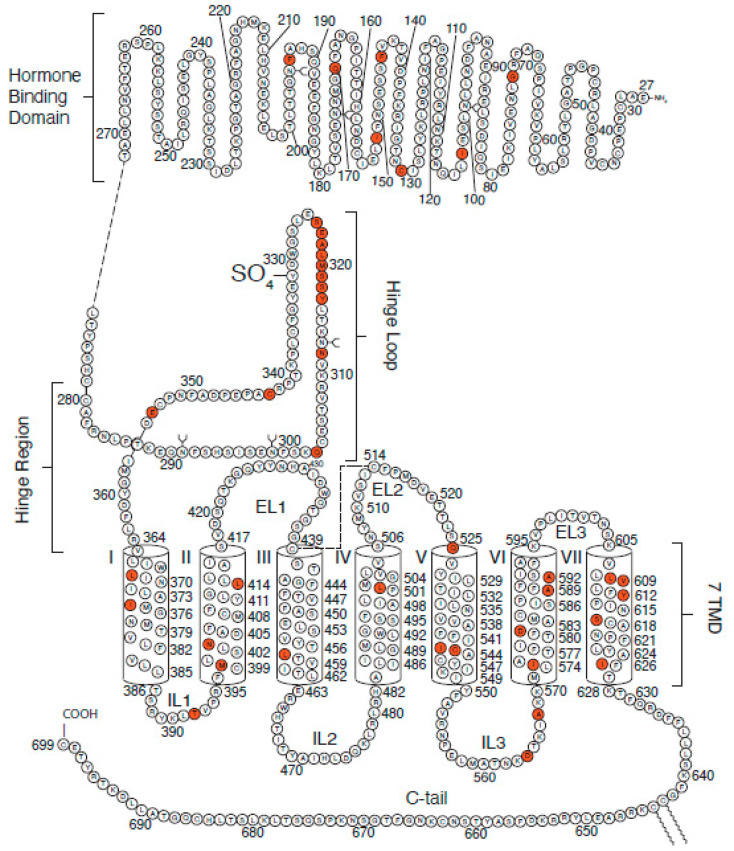
Structure of luteinizing hormone receptors (LHRs) showing the hormone-binding domain, the hinge region and the hinge loop, the seven transmembrane domains, and the intracellular C terminus. Mutations to amino acids shaded in red lead to LHRs loss of function.

LHRs are members of the GPCR superfamily and the GPCR subset of glycoprotein receptors (Figure 1). In response to the binding of either pituitary LH or placental hCG, receptor-associated G proteins located on the intracellular plasma membrane surface are activated, and intracellular levels of cyclic adenosine monophosphate (cAMP) increase. LHRs generally use G_s_ to activate adenylate cyclase, although, as shown by L. Birmbaumer’s group, phospholipase C is activated when LHRs or β adrenergic receptors are expressed at higher densities [9]. cAMP functions as an important second messenger within cells, activating signaling cascades that lead to hormone-mediated, receptor-specific cellular responses. The mature human LHRs (hLHRs) consist of 699 amino acids with a molecular mass of about 85,000 Da [1,5]. hLHRs are coded by a single copy gene that consists of 11 exons and 10 introns and are located on chromosome 2 p21 near the gene for FSHRs [10]. LHRs are synthesized as a single polypeptide chain that can be divided functionally into three domains [1,2,11]. The large N-terminus extracellular domain with 340 amino acids is extensively glycosylated and contains several leucine-rich repeats flanked by cysteine-rich regions and a hinge region. LH or hCG binds a single binding site in the LHR extracellular domain with high affinity [1,12]. The hinge region, located at the juncture between the extracellular domain and the seven transmembrane domains, links the receptor’s hormone binding region to its transmembrane domain [1]. Tyrosine 331, at the C-terminus of the hinge region, appears to be critical for human LH binding stability [5,13] and may play a role in differentiating signaling transduction resulting from hLHs or hCG binding to LHRs [13,14]. Deletion of exon 10 abolishes human LH but not hCG activity, a hormone-mediated response that is consistent with distinct intracellular responses triggered by LH or hCG binding to the extracellular domain [12,13,14,15].

Seven transmembrane α helices, connected by three extracellular loops and three intracellular loops, make up the transmembrane domain. Each α helical segment has about 25 amino acid residues [1,5], sufficient to span the plasma membrane lipid bilayer. Cysteine residues in the first and second extracellular loops that form disulfide bridges and the overall hinge region structure appear to stabilize the seven transmembrane α helices [16]. Conformational changes occurring in the seven transmembrane domains, after receptor activation by hormone binding, are translated across the membrane to the cytoplasmic membrane surface where LHRs activate G proteins and generate an intracellular signal.

The intracellular C-terminal region with 70 amino acids, together with intracellular loops, interacts with G proteins when LHRs are activated by hormone and helps initiate downstream signaling [1,2]. This domain has the most divergent functions of the three domains, regulating the trafficking of receptors from the endoplasmic reticulum to the plasma membrane as well as receptor internalization. It contains several threonines and serines that can be phosphorylated by protein kinase A in the intracellular pool [5,11]. For LHRs, phosphorylation of the receptor is not essential for desensitization of the receptor, the process that “turns off” LHRs’ response to hormones, and, unlike other members of the GPCR receptor family including rhodopsin, may not be essential to signal transduction [17].

### 2.2. Follicle Stimulating Hormone Receptors (FSHRs)

**Figure 2:**
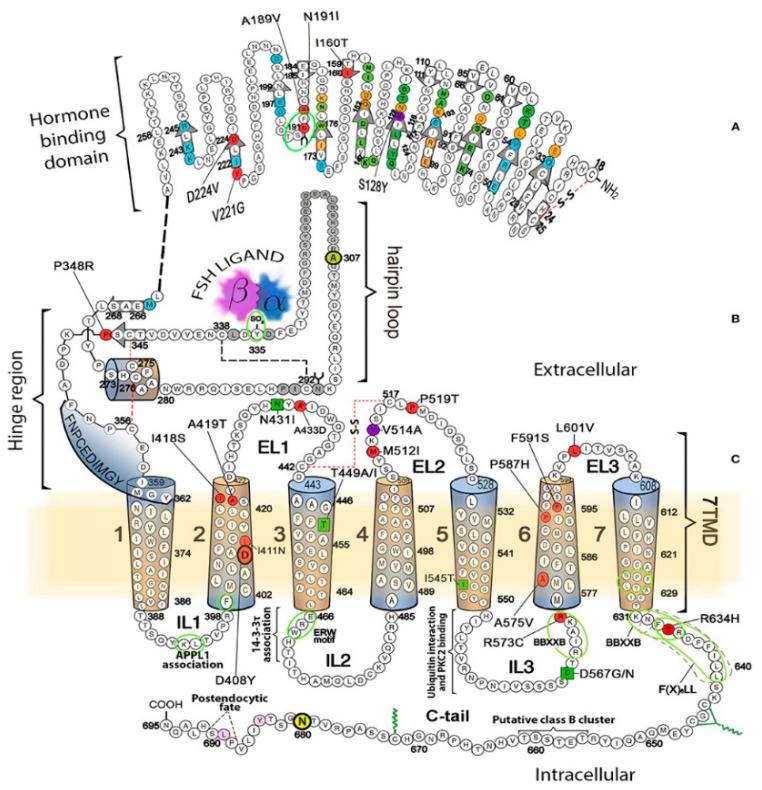
Structure of the follicle-stimulating hormone receptors (FSHRs) showing the hormone-binding domain, hinge region, seven transmembrane segments, and the intracellular C terminus. Mutations to amino acids shaded in red lead to the FSHRs loss of function [21]. This figure was reprinted from [21] under the terms of the Creative Commons Attribution 4.0 License.

The FSHRs are structurally similar to LHRs with a single polypeptide chain that folds to form an extracellular domain with leucine-rich repeats with a single binding site for FSH, seven membrane-spanning α helices linked by extracellular and intracellular loops and an intracellular domain (Figure 2). As is the case for LHRs, the hinge region, present at the junction between the extracellular domain and the start of the seven transmembrane domains, participates in transduction of signal resulting from binding of the ligand. A unique feature of FSHRs is proteolytic processing at the C-terminus before membrane expression [18]. Activation of receptor-associated G proteins initiates intracellular signaling pathways, including the production of cAMP by adenylate cyclase. The gene encoding the FSHRs lies near the gene for LHRs on chromosome 2p21–p16 and has 10 exons and 9 introns. The FSHR amino acid sequence produces a molecule of approximately 75 kDa molecule with glycosylation of *N*-linked sites adding to the receptor’s final molecular weight that ranges from 80–87 kDa [19].

Like LHRs, FSHRs are involved in the reproductive function in both males and females. In females, the maturation of follicles in the ovary requires active signaling by the FSHRs in response to FSH released from the anterior pituitary. Conversion of androgens to estrogens is initially upregulated by activation of the FSHRs and is subsequently regulated by signals from LH [20]. In males, the growth and maturation of Sertoli cells are under the control of FSH. Together with the LH effects on Leydig cell production of testosterone, both hormones, functioning through their respective receptors, are necessary for spermatogenesis in the testis. In addition to gonadal sites for FSHRs, the ovaries and testis, extra-gonadal sites of FSHRs have been identified with, as yet, unknown physiologic function [21].

## 3. LH, hCG, and FSH Structures

**Figure 3:**
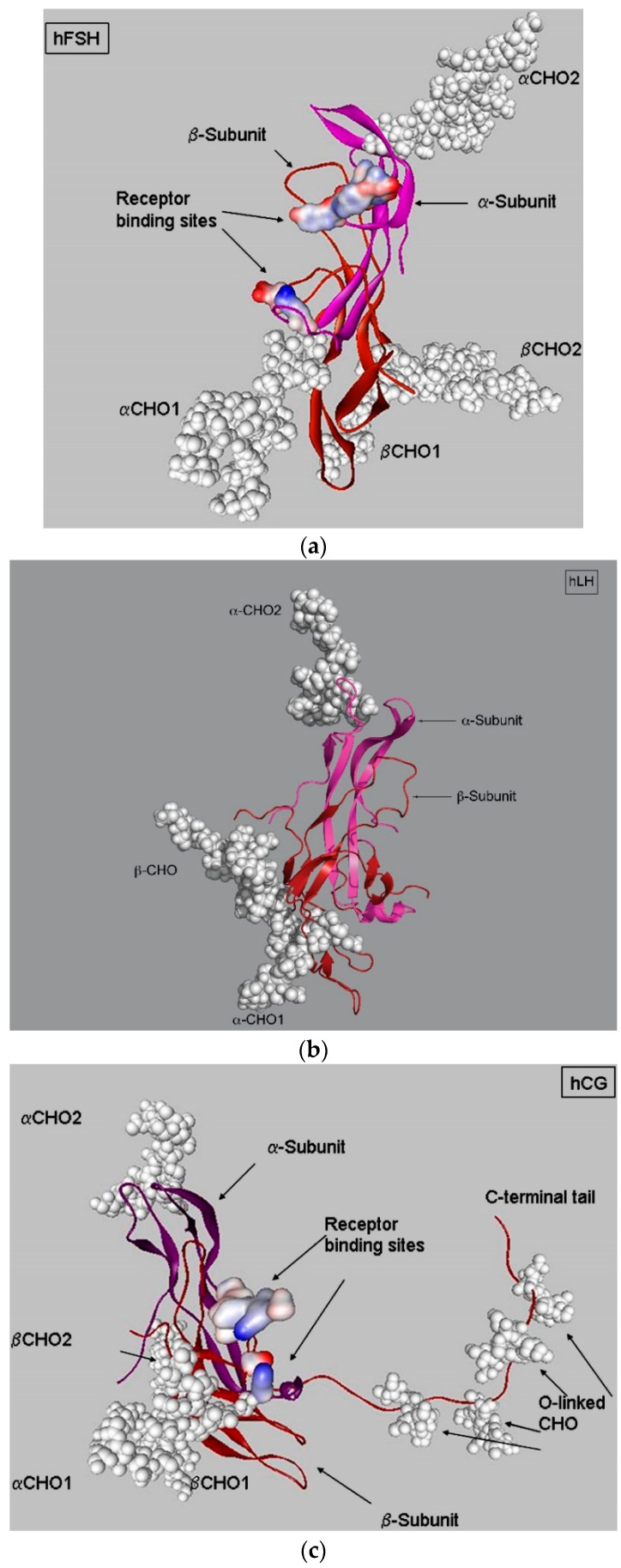
Structures of follicle-stimulating hormone (FSH) (**A**), and luteinizing hormone (LH) (**B**), and human chorionic gonadotropin (hCG) (**C**) showing the α and β subunits of each hormone. When compared to human LHRs (hLHs), the β subunit of hCG has additional amino acids and extensive glycosylation on the C-terminal tail that contribute to its higher molecular weight. FSH and hCG structures were from reprinted from [24] with permission from Elsevier. The figure of hLHs shown in the middle panel was provided by Dr. George Bousfield.

LHRs bind the pituitary-produced hormone, LH, and the placental hormone, hCG [2,22]. Pituitary LH, together with FSH, is synthesized and secreted by gonadotrophs located in the anterior lobe of the pituitary gland (Figure 3). Increased secretion of these hormones occurs in response to the activation of gonadotrophs expressing gonadotropin-releasing hormone receptors by a gonadotropin-releasing hormone from the hypothalamus.

LH, with a molecular weight of 28,500 Da [23], is a heterodimer comprised of the same α subunit found in hCG, LH, FSH, and thyroid-stimulating hormone and an LH-specific β subunit (Figure 3 middle panel) [24]. The α subunit has 92 amino acids with five disulfide bridges and an N-linked carbohydrate site [25,26] and is coded for by one gene located on chromosome 6 [27]. The β subunit of LH, coded by a single gene located on chromosome 19q13.3 [23,28], has 121 amino acids and contains six disulfide bridges and two N-linked carbohydrate sites [25,26]. There are reports of rare mutations in the LH β subunit that affect the secretion of the intact hormone, which, in males, causes delayed puberty, hypogonadism, and reduced circulating LH [29].

LH and FSH promote maturation of the follicle in the ovary and increase the production of estrogen [1,30]. In response to high levels of estrogen released during follicle development (folliculogenesis), a surge of LH from the anterior pituitary leads to the rupture of the pre-ovulatory follicle and ovum release [1,5]. The follicle then reorganizes to form the corpus luteum, which maintains secretion of estrogen and progesterone, both of which stimulate endometrial growth. In the absence of a pregnancy, the synthesis and secretion of estrogens and progesterone rapidly decrease, and the lack of hormonal support to the uterus leads to shedding of the endometrium [31]. If, however, there is a pregnancy, hCG, the hormone responsible for maternal recognition of pregnancy in humans, is synthesized by syncytiotrophoblast cells of the placenta. hCG sustains corpus luteum function by maintaining the production of progesterone during the first trimester of pregnancy. hCG, produced during pregnancy, also stimulates fetal Leydig cell production of testosterone [11], which, in turn, induces differentiation of the external male genitalia. In human males, human LH from the anterior pituitary drives testosterone production, which increases during puberty and post-puberty [1,32,33,34].

The hCG molecule has a molecular weight of approximately 36 kDa, which can vary depending on the extent of glycosylation [35]. It has the common α-subunit and a unique β-subunit connected by noncovalent interactions (Figure 3, right panel). Differences between the β subunits of LH and hCG are due to the expression of one of seven genes for the β subunit. The gene cluster for this subunit, localized on chromosome 19q13.3, contains seven homologous genes, one for *LH* β and six for the hCG β subunit. These hCG β genes appear to have arisen from *LH* β gene during primate evolution. The hCG β subunit has additional O-linked carbohydrate sites present on an additional 24 amino acids, which are not present on the LH β subunit (Figure 3, middle panel). Both the additional amino acids and glycosylation of those amino acids contribute to the higher molecular weight of hCG [35].

Both subunits, α and β, are required for the full biological function of LH and hCG. Although LH and hCG are very similar, LHRs can qualitatively discriminate between human LH and hCG through differences in hormone interactions with LHRs [13,36]. Furthermore, human LH and hCG may initiate different signaling pathways; hCG activates the cAMP pathway to a greater extent than human LH, while human LH preferentially activates extracellular signal-regulated kinases and protein kinase B pathways [22,36]. In addition to amino acid sequences, carbohydrates on both hormones are necessary for biological functions and hepatic clearance of the hormone from the body [37,38]. Most of the carbohydrates can be removed from hCG to generate deglycosylated forms of hCG [38], which binds LHRs with the same high affinity as the parent molecule, but with little or no function. This hormone antagonist has been found in serum from patients with chronic renal failure disease with accompanying hypogonadism [39].

As is the case for LH, hCG, and thyroid-stimulating hormone, FSH uses the common α subunit, which associates with a unique β subunit using noncovalent interactions and provides biological specificity (Figure 3, left panel). The FSH β subunit is encoded by a gene on chromosome 11 and expressed under the control of three groups of peptides, inhibins, activins, and follistatins, which modulate FSH β synthesis in both pituitary gonadotrophs and within the gonads. In general, inhibins and follistatins have indirect effects on β subunit expression by antagonizing activin activity. Their general regulation, as well as more specific roles in the ovary, are reviewed by Welt and Schneyer [40].

FSH is used in the treatment of both female and male infertility. FSH, either alone or in preparations containing hormone mixtures, is used to drive the development of multiple follicles in in vitro fertilization protocols that are customized to reflect women’s age and naturally occurring FSH levels [41]. In males, FSH, either alone or in combination with hCG, can be used to treat hypogonadotropic hypogonadism and stimulate spermatogenesis [42], although patient groups have been comparatively small and this pharmacologic use of FSH has varying degrees of efficacy [43]. 

## 4. Initiation of Signal Transduction: Effects of Receptor Aggregation on Receptor Function

Recent studies of GPCRs support the concept that receptor oligomerization is, for many members of the receptor family, required for receptor activation [44,45]. LHRs have been found clustered as dimers or oligomers in the plasma membrane (Table 1) and have generally been shown to undergo further clustering upon binding of a hormone. The cluster size of human LHRs which may reflect either larger groups of individual receptors or clustering of LHRs dimers or trimers, increases upon hormone binding in a concentration-dependent manner, and this may be involved in signaling, desensitization, and internalization of LHRs after activation by hormone [2,13,14,46,47]. 

It is important to note that, although dimerization and/or oligomerization of G protein-coupled receptors have become a well-accepted feature of G-protein coupled receptor signaling, a consensus on the organization of LHRs before and after the binding of ligand has not been reached. Using immunoprecipitation of epitope-tagged human LHRs, Tao et al. [48] showed that LHRs were self-associated in the absence of ligand and that there was an approximately 4-fold increase in receptor dimers or larger oligomers upon binding of a hormone. In contrast, Moyle and coworkers [49], using total internal reflectance imaging of the plasma membrane, did not find evidence for co-localization of fluorescence from individual receptors and, as a result, argued that LHRs were diffusely distributed on the membrane both before and after binding of the ligand. 

Unlike LHRs, FSHRs appear to be extensively aggregated before expression in the plasma membrane and do not undergo further measurable aggregation upon binding ligand (Table 1). In fluorescence correlation studies using a chimeric form of FSHRs with an LHR C-terminus to prevent cleavage of the C-terminus and preserve the receptor’s fluorescent tag, FSHRs diffused as a receptor dimer [50]. FSHRs results shown in Table 1 are also consistent with the appearance of dimers in the crystal structure of deglycosylated FSH coupled to the FSHRs exodomain and detection of these dimers in solution [51] and suggest that FSHRs, unlike LHRs, exist as a constitutive dimer independent of ligand binding.

**Table 1:** Survey of various experimental methods used to evaluate luteinizing hormone receptor (LHR) and follicle-stimulating hormone receptor (FSHR) clusters.

Receptor	Cell Type/Tissue	Experimental Method (s)	Probe (s)	Result	Ref.
Rat LHRs	Granulosa cells	Formaldehyde fixation/light microscopy/autoradiography	Rabbit anti-hCG/FITC-goat anti rabbit IgG	hCG treatment produced small LHR clusters at 4 °C, larger clusters at 37 °C	[52]
Rat LHRs	Rat luteal cells	Electron microscopy	Ferritin-LH (FE-LH)	FE-LH treated LHR clusters at 37 °C	[53]
Porcine LHRs	Porcine granulosa cells	Fluorescence resonance energy transfer (FRET) measured using spectrofluorimetry	FITC-/TrITC-hCG or FITC-/TrITC-hCG	Positive FRET (4 °C) for LH and hCG probes. LH: Reduced FRET (37 °C), hCG: minimal FRET (37 °C)	[54]
Rat LHRs	CHO cells	Fluorescence recovery after photobleaching (FRAP)	LHR-GFP (C-terminus)	LH reduced the fraction of mobile LHRs at 37 °C. hCG produced visible, immobile LHR clusters	[55]
Rat LHRs	CHO cells	Fluorescence recovery after photobleaching (FRAP)	LHR-GFP (C-terminus)	hCG increases LHR clusters which must dissipate before receptors can signal	[56]
		FRET	LHR-GFP/LHR-YFP	Immobile LHR clusters exhibit increased FRET	
Porcine LHRs	Porcine follicle membranes	Confocal microscopy	TrITC-hCG	Active: LHRs in small clusters Desensitized: LHRs in large clusters	[46]
		Time-resolved phosphorescence anisotropy	ErITC-hCG	Active: Small clusters, faster rotational correlation times Desensitized: larger clusters, slower rotational correlation times	
		FRET	FITC-hCG/TrITC-hCG	Active: Less FRET Desensitized: Increased FRET	
Human LHRs	HEK 293	Co-immunoprecipitation	c-myc-LHR (*N*-terminus)/ FLAG-LHR (*N*-terminus)	Coprecipitation of high molecular weight complexes from cells stably expressing LHRs. No detected change in complex molecular weight with hCG treatment.	[48]
Human LHRs	HEK 293	Fluorescence cross-correlation spectroscopy (FCCS)	hLHRs-delExon10–GFP/hLHR-C131R–mCherry; hLHR-K605E–GFP/hLHR-C131R–mCherry	FCCS showed cross-correlation for each receptor combination. Trans-activation partially rescued hCG response (increased cAMP) but not LH response	[13]
Rat LHRs	HEK 293	PALM super-resolution imaging	HA-WT-LHR, HA-LHR^B-^, FLAG-LHR^S-^ (HA.11/FLAG Abs)	WT alone and LHR^B-^ + LHR^S-^ exhibited intermolecular interactions favoring the formation of LHR oligomers	[57]
Human FSHRs	HEK 293	Imaging FRET	Anti-FSHR mAb-Alexa 588/Anti-FSHR An-Alexa 647	Positive FRET for untreated/FSH-treated FSHRs	[18]
		Co-immunoprecipitation	c-Myc-FSHR FLAG-FSHR	FSH oligomers form early in FSHR biosynthesis	
Human FSHRs		X-ray Crystallography	Asna^52^-FSH or fully glycosylated FSH	FSHRs are a functional trimer when binding Asna^52^-FSH	[58]
Human FSHRs	HEK293	Fluorescence correlation spectroscopy/photon counting histogram analysis	Chimeric human-FSHR with rat LHR C terminus-EGFP	Human FSHR/LHR C-terminus chimeras are homodimers	[50]

**Figure 4:**
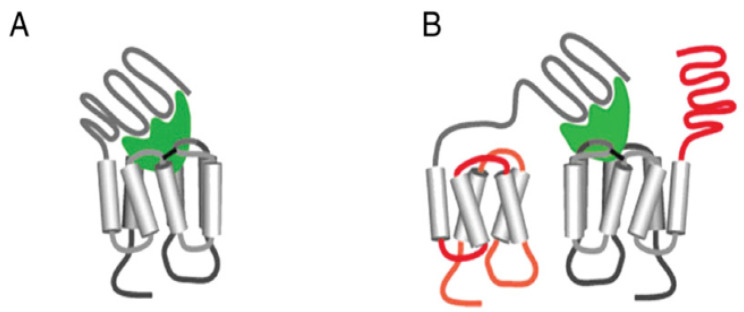
Schematic representation of cis-activation (**A**) and trans-activation (**B**) of a glycoprotein hormone receptor. Cis-activation occurs when hormone bound to the LHRs or FSHRs extracellular domain interacts with the portions of the same receptor involved in signal transduction. Trans-activation occurs when the hormone bound to the extracellular domain on one receptor interacts with an adjoining receptor that then initiates signal transduction. This figure was reprinted from [59] under the terms of the Creative Commons Attribution 4.0 License.

Initiation of LHRs or FSHRs signaling and, in the case of LHRs, receptor clustering, occurs upon binding of the ligand. The initial activation of LHRs and FSHRs occurs via two mechanisms. LHR-receptor-mediated signal transduction via *cis-activation* (Figure 4, panel A) involves high-affinity binding of either LH or hCG to the receptor’s exodomain and interactions between the hormone-occupied exodomain and the receptor endodomain. The receptor endodomain, the transmembrane domains together with the receptor’s C terminus, becomes activated and capable of interacting with G_s_. Ji and coworkers have reported another method by which LHRs may initiate signaling [59], receptor “trans-activation”, which involves two receptors (Figure 4, Panel B). A functional rat LHR exodomain binds ligand and then interacts with an adjoining receptor whose exodomain is incapable of binding the ligand. This leads to signal transduction by the second receptor’s functional endodomain, including activation of G-proteins and adenylate cyclase. LHR trans-activation can be shown [59] using cells transfected with LHRs containing one of several mutations in their exodomain that prevents the binding of the hormone. These cells produce cAMP in response to hCG treatment, presumably due to signaling through the competent LHRs endodomain. Using human LHRs, Grzelik et al. showed that co-expression of LHR with a deletion of Exon 10, a receptor capable of signaling but not binding ligand (LHR^B−^), together with an LHR signaling deficient mutant (LHR^S−^), produces partial rescue of signaling in vitro [14]. In transgenic mice, co-expression of LHR^B−^ and LHR^S−^, restore reproductive function [60]. FSHRs are also capable of trans-activation, which Ji and coworkers have demonstrated using a strategy similar to that used for LHRs [47]. FSHR trans-activation, biased signaling, and receptor oligomerization have been recently reviewed by Szymariska et al. [61]. Implied in either LHR or FSHR trans-activation is that a hormone-occupied glycoprotein receptor exodomain (R^S−^) capable of rescuing signal transduction by a receptor that is unable to bind ligand (R^B−^) is that R^S−^ and R^B−^ must come into close proximity to initiate trans-activation [60].

Whether a G protein-coupled receptor is activated by cis- or trans-activation may affect the selection of signaling pathways. FSH receptors can signal via either cAMP or phosphatidyinositol following cis-activation but through only one or the other pathway with trans-activation [62]. The selection of a signaling pathway, however, may also arise from the activation of multiple signaling pathways within a target cell as, for example, occurs when there is crosstalk between signaling pathways used by a GPCR and those used by other receptors [61]. 

## 5. LHRs and FSHRs in Disease

Mutations in FSHRs affect fertility in women (Table 2). These mutations cause disrupted receptor function ranging from misfolding errors that limit receptor expression in the plasma membrane to mutations in plasma membrane expressed receptors that affect ligand binding or signaling transduction [21]. Compromised FSHRs function due to the presence of gene variants is associated with premature ovarian failure and amenorrhea [63]. 

As with FSHRs, mutations in LHRs (Table 2) can affect the trafficking of the receptor to the plasma membrane and produce disorders in fertility, similar to those seen with the expression of inactivating receptor mutations [64]. Because of the role of LHRs in male gonad development, males expressing inactivating LHR mutations can exhibit micropenis, hypospadias, delayed puberty, and Leydig cell hypoplasia with lower LHR expression. In some forms of XY disorders of sexual development (XY DSD), including but not exclusive to inactivating mutations of LHRs, incomplete masculinization in utero can result in female external genitalia but, at ages associated with puberty, the absence of breast development and primary amenorrhea. Inactivating mutations of LHRs in women can result from disruptions in the trafficking of LHRs to the plasma membrane and reduced plasma membrane expression. Women exhibit primary amenorrhea despite the presence of secondary sex characteristics and, depending on the severity of the mutation, impaired fertility due to empty follicle syndrome.

Mutations in specific LHR domains can cause LHRs to be constitutively activated, leading to precocious puberty in males [65,66,67,68,69]. Activating LHR mutations in Table 2 are indicated by an *. Shenker’s group was the first to identify an LHR mutation associated with familial male-limited precocious puberty [66] and further showed that expression of this receptor in an unrelated cell type resulted in markedly increased intracellular cAMP in the absence of available LH or hCG. Clinically, patients with familial male-limited precocious puberty, which occurs for a number of LHR mutations, can present with puberty as early as 4 years of age [66], Leydig cell hyperplasia, high levels of testosterone, and low levels of gonadotropins [67].

The role of LHRs and FSHRs in polycystic ovary syndrome (PCOS) merits additional discussion. PCOS is itself, a heterogeneous disease with various diagnostic criteria [70]. PCOS affects fertility in upwards of 20% of women with clusters in families [70]. Although PCOS is associated with basal and glucose-mediated hyperinsulinemia, there is also limited evidence for glycoprotein hormone receptor involvement. Zou and colleagues have described LHR gene polymorphisms associated with PCOS [71]. Similarly, the N680S FSHR mutation has been identified in patients with PCOS, as well as women with premature ovarian syndrome, which is defined as ovarian failure in women before the age of 40 [72,73].

There are also isolated reports of FSHR or LHR expression in cancers associated with reproductive tissues. Both FSHRs and LHRs have been identified in ovarian cancers [74]. There may be therapeutic value to identifying FSHR expression in ovarian cancers as has been demonstrated in patient-derived tissues; Perales-Puchalt et al. suggest that FSHR-expressing T cells may have utility in immunotherapy regimes that increase T cell targeting to ovarian cancer cells [75], a strategy that may be valuable in extending the relapse-free interval in ovarian cancer. Lenhard et al. examined the outcomes of patients with ovarian cancer expressing both FSHRs and LHRs and showed that FSHR expression was associated with poorer disease outcomes [76]. Over half of the patient samples examined expressed FSHRs, and for those patients expressing FSHRs alone, prognosis was worse than for patients expressing only LHRs. This result was in agreement with previous studies showing that FSH promotes tumor cell growth [77], an effect opposed by LH. FSHRs have also been described in prostate cancers, as well as in normal prostate tissues and tissues from patients with benign prostatic hypertrophy [78], and the diagnostic and therapeutic utility of this expression in prostate disease has been reviewed recently [79]. More generally, FSHRs have been found in tumor epithelial cells or the vasculature of solid tumors or sarcomas where the receptor-expressing vessels provide a link between tumors and the general circulation (reviewed in [80]). 

There are comparatively few studies that directly examine LHR and FSHR function in human diseases not linked to reproductive tissues. In rodents, FSHR knockout produces depression-like behaviors and oxidative stress in the brain [81]. LHR knockout during murine development affects retinal vascularization and reduces levels of vascular endothelial growth factor [82], while in mature mice, LHR knockout reduces the β-amyloid peptide load found in Alzheimer’s Disease [83]. Increased circulating levels of LH, presumably functioning through LHRs, have been implicated in higher rates of Alzheimer’s Disease in postmenopausal women compared to men [67], as well as skin changes in anovulatory and postmenopausal women [84,85,86]. Increased FSH levels have been linked to decreased insulin resistance in older, postmenopausal women [87], although the relationship between insulin resistance and the number of available FSHRs was not evaluated.

**Table 2:** Mutations in FSHRs and LHRs associated with altered receptor function and pathology.

Receptor/Mutation	Homozygous/HeteRozygous	Phenotype	Reference
Follicle Stimulating Hormone Receptor			
S128Y(T)		Spontaneous ovarian hyperstimulation syndrome during pregnancy, increased hCG, TSH response	[88]
I61N	Heterozygous	Amenorrhea, infertility, early antral follicles, no cAMP	[89]
T449A		Spontaneous ovarian hyperstimulation syndrome during pregnancy, increased hCG, TSH response	[90]
T449I		Spontaneous ovarian hyperstimulation syndrome during pregnancy, increased hCG, TSH response	[90]
P519T		Failure of FSH to bind to FSHRs, hypergonadism	[91]
D567N		Spontaneous ovarian hyperstimulation syndrome during pregnancy, increased hCG, TSH response, impaired FSHR desensitization, hypogonadotropic hypogonadism, precocious pseudopuberty	[90]
N680S	Homozygous	PCOS, premature ovarian syndrome, high circulating FSH, decreased FSHR activity	[92]
P688T	Heterozygous	Amenorrhea, infertility, early antral follicles, decreased cAMP	[63]
**Luteinizing Hormone Receptor**			
L10P		Signal peptide mutation causing micropenis, cryptorchidism	[93]
Q18-L19ins9		Signal peptide mutation causing severe Leydig cell hypoplasia	[94]
I114F	Heterozygous	XY disorder of sexual development (XY DSD), Leydig cell hypoplasia, decreased LHRs, reduced signal transduction	[95]
C131R	Homozygous	Impaired cAMP response, micropenis, hypospadias, Hypoplastic phallus with hypospadias, XY DSD	[96]
V144F	homozygous	XY DSD	[97]
I152T		No Leydig cells, immature seminiferous tubules, impaired hormone binding, signal, genitalia with some virilization	[98]
Q170Stop	Homozygous	Nonsense mutation causing primary amenorrhea	[99]
F194V		XY DSD, no cAMP signal	[100]
N312S	Homozygous	Leydig cell hypoplasia in males and higher success rates for IVF pregnancy in females	[101]
Deletion between Y317 and S324	Homozygous	Hypergonadism in males and Primary and secondary amenorrhea in females	[102]
Y317-S324 deletion	Homozygous	Males: Splice site mutation causing micropenis, delayed puberty, oligospermia Females: infertility with/without oligomenorrhea in females	[64]
C343S	Compound heterozygote	XY DSD	[103]
E354K	Homozygous	XY DSD, undescended testes in males and primary amenorrhea in females	[104]
L368P		Missense mutation causing precocious puberty, increased cAMP *	[105]
I374T	Heterozygous	XY DSD, Leydig cell hypoplasia	[106]
T392I	Double homozygote	XY DSD, Leydig cell hypoplasia	[106]
M398T	Heterozygous	Familial male limited precocious puberty *	[107]
N400S	Homozygous	Infertility, empty follicle syndrome	[108]
I415T	Heterozygous	Leydig cell hypoplasia, micropenis, no cAMP production	[109]
L457R		Elevated cAMP, precocious puberty *	[12]
T461I, exon 6A mutation	Compound heterozygote	XY DSD	[110]
L502P		XY DSD, Leydig cell hypoplasia	[111]
Q525Stop	homozygous	Primary amenorrhea	[99]
I528Stop	Heterozygous	Leydig cell hypoplasia	[112]
I542L		Familial male limited precocious puberty *	[67]
C543R	Compound heterozygote	XY DSD	[103]
C545Stop	Heterozygous	No cAMP, XY DSD	[113]
R554Stop	Homozygous	Males: XY DSD Females: small uterus, cystic ovary, primary and secondary amenorrhea	[114]
D564G	Heterozygous	Familial male limited precocious puberty *	[115]
A568V	Homozygous	Precocious puberty *	[105]
I575L	Heterozygous	Familial male limited precocious puberty *	[107]
D578G (H, E, Y)		Familial male precocious puberty, Leydig cell hyperplasia, precocious puberty *	[65,66,116,117,118,119]
A593P	Homozygous	XY DSD, Leydig cell hypoplasia in males and primary amenorrhea, lack of breast development, infertility in females	[96]
I625K	Homozygous	Micropenis, no puberty, infertility	[96]
Exon 8 and S616Y deletion	Compound heterozygote	Leydig cell hypoplasia, micropenis, hypospadias	[120]
Exon 10 deletion	Homozygous	Hypogonadism, no puberty	[121]

* Phenotypes identified with an asterisk have LHRs with activating mutations.

## 6. Turning the Signaling by Membrane-Expressed LHR Receptors on or off 

**Figure 5:**
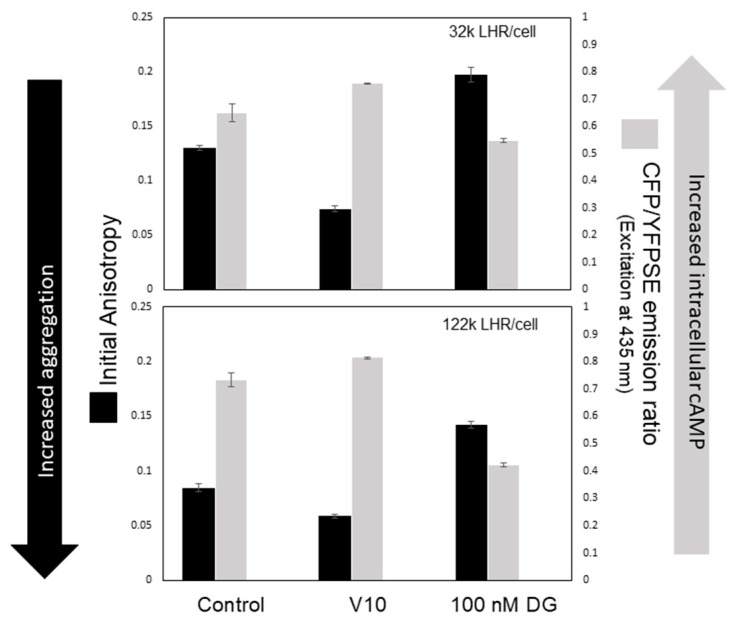
Comparison of the extent of LHR aggregation in cells expressing physiologically relevant receptor numbers (32 k LHR/cell) and cells overexpressing LHRs (122 k LHR/cell). The extent of receptor aggregation was measured as a function of receptor anisotropy (Y-axis, left). Decreased anisotropy values indicate more extensive clustering of the receptor, as shown by the arrow. The extent of receptor clustering was compared to intracellular cAMP levels (Y-axis, right) measured using a cAMP probe. In some experiments, cells were pre-treated with decavanadate (V10), which reduced the extent of membrane lipid packing (data not shown) while causing increased clustering of LHRs and increased cAMP [125]. Pre-treatment of cells with 100 nM deglycosylated hCG (DG), reduced receptor clustering, and reduced cAMP signaling. Both homo-FRET results and intracellular cAMP levels are expressed as mean ±SEM of 30 measurements for each condition. Statistical evaluation of mean differences in untreated and treatment groups was analyzed by one-way ANOVA followed by the Tukey multiple comparison test and Student’s *t*-test to compare between two groups using R version 3.3.1. *p*-values < 0.05 were statistically significant.

The ability to regulate LHR or FSHR signaling, particularly in pathological conditions involving receptors that contain either activating or inactivating mutations, is of considerable pharmacologic interest. Our group has explored aspects of this problem using biophysical methods and has addressed three problems. The first was to better evaluate conditions under which hLHRs signal in the absence of a bound ligand. Recent work using stable cell lines expressing physiologic numbers of hLHRs per cell (32 k LHR/cell) or overexpressing the receptor (122 k LHR/cell) suggests that constitutively active receptors produce maximum levels of intracellular cAMP [122] particularly when receptors are expressed at high numbers when compared to otherwise untreated control cell populations (Figure 5). Thus, the extent of receptor aggregation is associated with receptor density on the cell surface; when there are comparatively few receptors on the cell surface, LHRs will aggregate in response to certain stimuli including hCG, whereas overexpressed LHRs are extensively aggregated and undergo little additional change in their aggregation state regardless of treatment. Anisotropy is a measure of the extent of receptor aggregation, and with increasing aggregation, values for anisotropy become smaller. When compared with cells expressing 32 k LHR/cell with cells that overexpress LHRs, there are significantly lower anisotropy values for overexpressed receptors, 122 k LHR/cell (lower panel) accompanied by significant increases in intracellular cAMP. Thus, when the LHRs are more extensively aggregated, intracellular cAMP levels are higher, and signaling has occurred in the absence of hormone.

The second problem was to evaluate whether changes in receptor aggregation, specifically an increase in the apparent aggregation of LHRs, was sufficient to drive increased LHR signaling. To this end, we needed to identify a treatment or agent that facilitated LHR aggregation. Although the molecular details of this signaling mechanism are not well understood, we hypothesized that inhibitors of protein tyrosine phosphatases and signal transduction may be useful in this regard as reported for other membrane receptors [123,124]. We tested selected vanadium compounds that are known protein phosphatase inhibitors and found that it was possible to activate LHRs without ligand, using vanadium compounds to treat cells [122,125,126,127,128] The initially tested vanadium compounds were coordination complexes known to interact with membranes [129] or, in some cases, to penetrate the membrane interface [129,130]. These vanadium-containing compounds included bismaltolato oxidovanadium (IV) [131] and dipicolinato *cis*-dioxovanadium (V) [132,133]. More recently, we investigated vanadium complexes, including large oxovanadates, that were not able to penetrate the membrane and identified similar effects on LHR clustering. The effects of V_10_ are shown in Figure 5. From other work, it appears that the addition of this compound to the growth media causes an interaction with the cell membrane lipids located in the bulk plasma membrane and results in the reduction in lipid order [125,133]. Decreased lipid order causes the redistribution of LHRs from the bulk membrane to specialized membrane microdomains where LHRs exhibit significantly reduced anisotropy, evidence of receptor clustering, and signals despite the absence of ligand, as seen for cells overexpressing LHRs (122 K LHR/cell). The role of tyrosine phosphorylation of LHR aggregation, signaling, or receptor desensitization cannot be evaluated, as has been discussed previously [122]. Nevertheless, these results suggest that plasma membrane lipids may be a useful target for pharmacologic agents designed to activate GPCRs that are expressed in the plasma membrane and use plasma membrane rafts to signal [134]. We should, however, point out that this is not the only strategy that can be used to successfully activate LHRs. Newton et al. [135] used small molecules functioning as chaperones (L-CHAP) to increase LHR expression on the plasma membrane, a useful strategy for rescuing receptor function when LHR mutations disrupt membrane expression. Interestingly, this exposure to L-CHAP has, in some cases, also restored at least partial responsiveness to hCG.

A critical problem, from a pharmacologic perspective, is how to reduce signaling by constitutively-active LHRs that are expressed in the plasma membrane and concentrated in membrane rafts in the absence of a hormone [136]. Constitutively active receptors are expressed, as an example, in precocious puberty, as discussed above [66]. Interestingly, one strategy for reducing LHR cluster formation and receptor-mediated signaling using an hCG antagonist, deglycosylated hCG (DG) (Althumairy, unpublished results). DG can be produced synthetically but has also been identified in serum from male patients with chronic renal failure and hypogonadism [39]. We have used DG, prepared by Dr. George Bousfield [137,138], that binds LHRs with the same affinity as hCG but exhibits little or no ability to stimulate cAMP levels within cells [139,140]. As shown in Figure 5, when compared with values obtained using untreated cells, DG significantly increased fluorescence anisotropy in CHO cells, stably expressing either physiologically relevant numbers of receptors or over expressing LHRs. In addition, decreases in LHR aggregation were accompanied by significant decreases in intracellular cAMP levels. These results suggest that DG treatment may be uniquely effective in reducing both receptor clustering and the signal associated with constitutively active receptors. It is also important to recognize that receptor clustering and the signal resulting from various extents of clustering in vivo do not occur in isolation from signaling events mediated by other receptors present on the membrane, including GPCRs utilizing clathrin-coated pits or β-arrestin. Simultaneous active signaling by non-LHR receptors may modulate LHR receptor signaling [141] in a process that remains poorly understood.

## 7. Conclusions

Diseases involving the glycoprotein hormone receptors, LHRs or FSHRs, are difficult to both identify and treat. Such diseases can result from under or overexpression of receptors, receptor mutations that affect membrane expression, control of receptor-mediated signaling, or the appearance of receptor activity in tissues that are normally not associated with LHR or FSHR function. In addition to identifying the underlying causes of such diseases, not a trivial process, strategies for treatment are limited. Thus, using, for example, modified hormones to disperse clustered receptor, targeting the cell membranes to drive mutant receptor signaling in raft domains, or manipulating the oligomers formed upon hormone binding to LHRs or FSHRs, may prove useful in directing research exploring pharmacologic treatments designed to improve fertility or provide more regulated cell growth.

## Abbreviations

LHR	Luteinizing hormone receptor
FSHR	follicle-stimulating hormone receptor
GPCR	G protein-coupled receptor
LH	luteinizing hormone
FSH	follicle-stimulating hormone
hCG	human chorionic gonadotropin
DG	deglycosylated human chorionic gonadotropin
COS	polycystic ovary disease
